# A Case of Cocaine-Induced Acute Liver Failure Reversed With N-Acetylcysteine

**DOI:** 10.7759/cureus.41579

**Published:** 2023-07-08

**Authors:** Mary C Mitchell, Cleon Rogers

**Affiliations:** 1 Medical School, Edward Via College of Osteopathic Medicine, Auburn, USA; 2 Internal Medicine, Christ Health Center, Birmingham, USA

**Keywords:** ischemic hepatitis, severe hepatic encephalopathy, n-acetylcysteine, cocaine-induced ischemic hepatitis, acute liver failure (alf)

## Abstract

Acute liver failure (ALF) is a life-threatening injury that is most often caused by drug-induced injury, including acetaminophen overdose, in the United States. The hallmarks of ALF are hepatic encephalopathy and coagulopathy in a patient without an established history of liver disease. While acetaminophen overdose has an antidote, that is N-acetylcysteine (NAC), when given acutely, most other causes of hepatic failure require an urgent liver transplant. In this paper, we report a case of cocaine-induced acute liver failure that was reversed with the administration of NAC. Our case began when a middle-aged male presented to the emergency department complaining of nausea, vomiting, fatigue, and confusion for the past three days. His past medical history was pertinent for a history of opioid use disorder and his physical exam was remarkable for somnolence, asterixis, and periumbilical ecchymoses. His initial lab results showed markedly elevated liver function tests, prolonged coagulation studies, and a urine drug screen that was positive for cocaine. During the patient’s interview, his vital signs became unstable. He was intubated for airway protection and transferred to a tertiary care facility for liver transplant evaluation with the diagnosis of cocaine-induced acute liver failure. There he received NAC, lactulose, rifaximin, and vasopressors. On day two of treatment, his clinical condition greatly improved, and he was extubated. He continued to receive NAC until day five when his liver function tests and coagulopathy improved enough to stop treatment. This case report highlights the clinical benefit of NAC in a case of cocaine-induced acute liver failure, improving the patient’s survival and eliminating his need for a liver transplant.

## Introduction

Acute liver failure (ALF) is a life-threatening injury to the liver that occurs rapidly in patients without a history of liver disease [1]. Its hallmarks are increased international normalized ratio (INR) and hepatic encephalopathy [2]. Patients often present with fatigue, nausea, vomiting, and altered mental status. ALF may be drug-induced, infectious, or ischemic in origin. In the United States, ALF is most commonly due to drug-related injuries, including acetaminophen overdose [2]. However, viruses such as hepatitis A and B, herpes simplex virus (HSV), cytomegalovirus (CMV), and Epstein-Barr virus (EBV) may present with similar symptoms. Urea cycle disorders like Wilson's disease and obstructive disorders like Budd-Chiari syndrome are rare causes of ALF [2].

The severity of hepatic encephalopathy is a prognostic indicator in ALF and acute changes warrant management in a tertiary care center [2]. Hepatic encephalopathy is a neurological manifestation of elevated levels of multiple neurotoxins in the blood, most notably ammonia. The liver is the organ responsible for ammonia metabolism. When the liver is dysregulated, ammonia builds up in the blood. This most often occurs in patients with liver cirrhosis due to decreased blood flow through the liver attributable to portosystemic shunts [3]. However, this can also occur in a hypoperfused liver, or a liver undergoing shock. This reduction in hepatic blood flow causes decreased metabolization of ammonia into its biologically excretable conjugate, ammonium. Therefore, there is increased ammonia in the blood and in the cerebrovascular system, causing the hallmark signs of asterixis (hand flapping), incoordination, myoclonus, cognitive impairment, and eventually, if severe and left untreated, coma [4]. The treatment of choice for hepatic encephalopathy is a combination of lactulose and rifaximin or neomycin [3,4]. Lactulose is an osmotic laxative that increases the intra-intestinal conversion of ammonia to ammonium [4]. Rifaximin and neomycin are antibiotics that work to limit intestinal bacterial production of ammonia in the gut [4]. Therefore, in patients with suspected hepatic encephalopathy, it is important to start lactulose and rifaximin or neomycin to decrease the blood ammonia level as this has been shown to decrease mortality [3]. 

Acute liver failure is divided into three clinical acuity groups based on the onset of hepatic encephalopathy after the appearance of jaundice. Hyperacute liver failure is defined as the appearance of hepatic encephalopathy less than a week after the initial presentation of jaundice. Acute liver failure is the presentation of hepatic encephalopathy in less than a month from the initial presentation of jaundice and subacute liver failure is the presentation of hepatic encephalopathy less than three months from the presentation of jaundice [5]. Clinically, hyperacute liver failure is correlated with a worse prognosis and requires immediate medical intervention including administration of N-acetylcysteine, intensive care unit supportive therapy, and liver transplant, depending on etiology [5]. 

The mainstay of treatment for ALF depends on the etiology. N-acetylcysteine (NAC), a drug that replenishes glutathione in the liver, is used to reverse acetaminophen toxicity, while a rapid liver transplant is required in most other cases [6]. While NAC has been shown to greatly improve other non-acetaminophen causes of acute liver failure, a recent meta-analysis concluded that NAC should not be given priority over liver transplant in patients with non-acetaminophen forms of acute liver failure [7]. This recommendation stems from the finding that NAC does not improve survival in non-acetaminophen acute liver failure when compared to placebo [7]. The most important factor that determines if a patient is a candidate for a liver transplant is the presence of pre-existing liver disease, such as cirrhosis [5]. This is determined through a liver ultrasound or computed tomography (CT) scan. Hyperacute liver failure patients may have normal-appearing livers or hypoechoic livers with necrosis that may mimic hepatic steatosis on CT [5]; whereas patients with chronic liver failure or acute-on-chronic liver failure will have a nodular liver on imaging studies [5]. It is important to differentiate these pathologies with imaging studies, as it influences management and prognosis. Patients without cirrhosis are placed at a high priority for liver transplant and have a better-predicted prognosis than patients with cirrhosis that are lower on the transplant list and managed supportively [5].

## Case presentation

A middle-aged man presented to the emergency department complaining of nausea, vomiting, fatigue, and confusion for the past three days. The patient was somnolent upon exam, limiting the interview. His past medical history was insignificant, but his social history was notable for a 20+ year history of oxycodone use and a history of intravenous (IV) drug use. His home medications included methadone daily for opioid use disorder and albuterol for asthma as needed. His vital signs upon admission were notable for a temperature of 36.9 degrees Celsius, heart rate of 64 beats per minute, respiratory rate of 14, blood pressure of 135/61 mm Hg, and oxygen saturation of 93% on a 2-liter nasal cannula. The patient was somnolent on physical exam, but arousable to voice and was oriented to person, place, and time. Asterixis and mild periumbilical ecchymoses were also found; the remainder of the physical exam was unremarkable. 

The differential diagnosis after initial examination included the following: acute stroke, sepsis, diabetic ketoacidosis, acetaminophen overdose, illicit drug overdose, viral hepatitis, autoimmune hepatitis, acute kidney injury, acute pancreatitis, alcohol intoxication, thyroid storm, Wilson’s disease, acute presentation of the human immunodeficiency virus (HIV), Epstein-Barr virus (EBV), cytomegalovirus (CMV), herpes simplex virus (HSV), or syphilis. Thus, a wide variety of labs were ordered including a complete blood count (CBC), comprehensive metabolic panel (CMP), coagulation studies, thyroid stimulating hormone (TSH), thyroxine (T4), copper level, aspirin level, acetaminophen level, blood alcohol level, creatinine, urinalysis, urine culture, blood culture, hepatitis panel, rapid plasma reagin (RPR), virology studies for HIV, EBV, CMV, and HSV, and immunology studies for antinuclear antibody (ANA), anti-mitochondrial antibody (AMA), anti-smooth muscle antibody (ASMA), cytoplasmic anti-neutrophil cytoplasmic antibody (c-ANCA), and perinuclear anti-neutrophil cytoplasmic antibody (p-ANCA). 

His initial lab results were remarkable for an elevated white blood count, alanine transaminase (ALT), aspartate aminotransferase (AST), creatine kinase (CK), and ammonia (Table 1). Coagulation tests showed a prolonged prothrombin time (PT) of 22.5 and an international normalized ratio (INR) of 2.12. His kidney, thyroid, and copper studies were within normal limits. Serology tests were negative for aspirin, acetaminophen, and alcohol. However, urine toxicology was positive for benzodiazepines, oxycodone, methadone, and cocaine. Urinalysis was positive for red blood cells, white blood cells, 2+ protein, 2+ blood, and 3+ leukocyte esterase. Urine and blood cultures were both negative. The hepatitis panel did not reveal any acute or chronic infections of hepatitis A, B, or C. Additional lab studies were negative for HIV, EBV, CMV, HSV, and syphilis. Immunology work-up was also negative for ANA, AMA, ASMA, c-ANCA, and p-ANCA. 

**Table 1:** Initial laboratory results ALT: alanine transaminase, AST: aspartate aminotransferase, CK: creatine kinase

Laboratory results	Measured value	Standard value
White blood count	17 x 10^3^/cmm	4.0-11.0 x 10^3^/cmm
ALT	6101 U/L	7-52 U/L
AST	3795 U/L	12-39 U/L
Alkaline phosphatase	114 U/L	37-117 U/L
Total bilirubin	1.05 mg/dL	0.3-1.4 mg/dL
Ammonia	192 mcMol/L	12-60 mcMol/L
CK	1448 U/L	55-170 U/L

CT of the head, chest X-ray, electrocardiogram (EKG), and transthoracic echocardiogram were within normal limits. However, an ultrasound of the abdomen revealed hepatomegaly and hepatic steatosis. A CT of the abdomen was performed, which confirmed this finding and also revealed evidence of pancreatitis. 

Based on the patient’s normal lab and imaging results, we were able to exclude the following diagnoses: acute stroke, acute intracranial pathology, acetaminophen overdose, viral hepatitis, diabetic ketoacidosis, autoimmune hepatitis, alcohol intoxication, thyroid disease, Wilson’s disease, acute viral illness, and malignancy.

During the interview, the patient’s mental status decreased, he became tachycardic and he began to have low blood pressure. The patient was intubated for airway protection, started on lactulose, and transferred to a tertiary care facility the same day for liver transplant evaluation. The patient’s rapidly declining clinical picture, lab results, and imaging studies suggested acute liver failure caused by cocaine ingestion.

**Figure 1:**
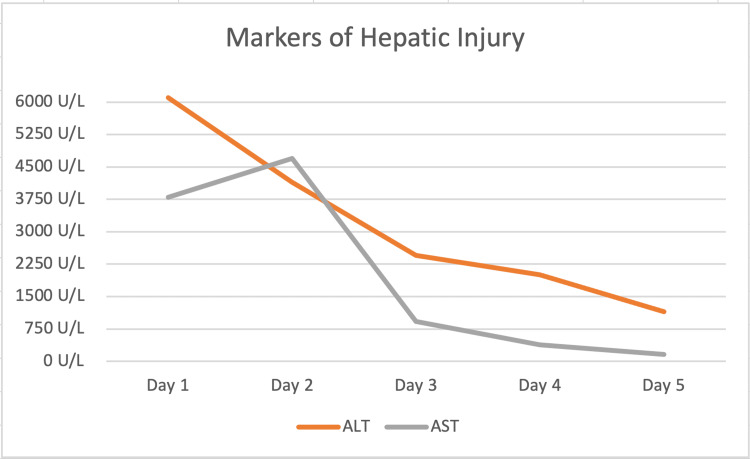
ALT and AST measurements over the course of the patient’s hospital stay ALT: alanine transaminase, AST: aspartate aminotransferase

**Figure 2:**
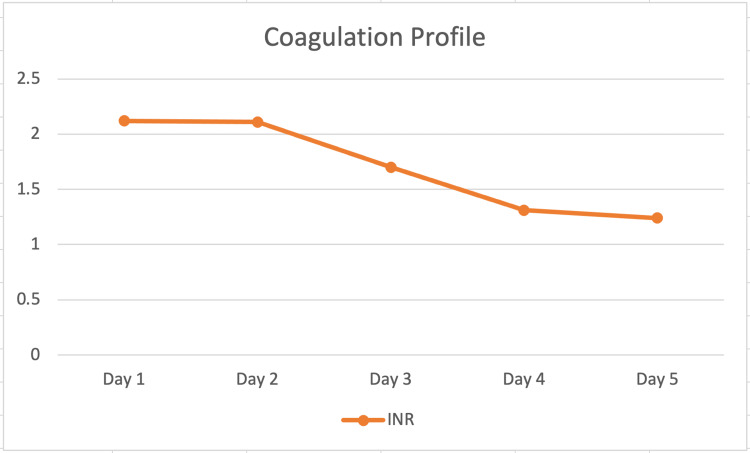
INR measurements over the course of the patient’s hospital stay INR: international normalized ratio

In the tertiary facility, the patient was managed with the standard dose of lactulose, NAC, rifaximin, and vasopressors, while awaiting transplant evaluation. On day two of hospitalization, the patient’s clinical condition improved, and he was extubated and placed on 2 liters of oxygen on a nasal cannula, eliminating his need for transplant evaluation. The patient continued to receive NAC, lactulose, and rifaximin. Over the course of the next five days, the patient’s liver function tests (Figure 1) and coagulation profile (Figure 2) greatly improved. The patient was discharged after five days of hospitalization with improved labs showing ALT of 1153 U/L, AST of 164 U/L, and INR of 1.24. The patient was discharged on lactulose for 30 days, pantoprazole for 60 days, and methadone according to his home regimen and was instructed to follow up in an outpatient facility for labs in one week.

## Discussion

It is well-established that cocaine has the potential to cause multi-organ system dysfunction including cardiac damage, systemic vasoconstriction, and hepatotoxicity [8]. Cocaine causes cardiac dysfunction through three main mechanisms: blockage of K+ and Na+ channels leading to ischemia, increased norepinephrine levels causing vasoconstriction of the coronary arteries, and calcium overload damaging cardiomyocytes [8]. Cocaine also affects systemic vasculature by alpha-1-mediated vasoconstriction and disruption of endothelial cell nitric oxide release, limiting vasorelaxation [8]. Therefore, patients with acute cocaine intoxication commonly present with signs of cardiac hypoperfusion and ischemia, such as chest pain and myocardial infarction [9].

Similarly, cocaine may cause hepatocyte damage through two mechanisms: hypoperfusion and oxidative stress. It has been suggested that cocaine reduces hepatic blood flow, causing shock liver, or ischemic hepatitis [10,11]. The findings of shock liver have been associated with cocaine-induced hepatitis in several case reports [9, 11-13]. Cocaine-induced ischemic hepatitis is consistent with the features in our case, as the patient’s condition progressed to hypotension and tachycardia, requiring vasopressors. In addition to hypoperfusion, cocaine causes hepatotoxicity and oxidative stress through its metabolite, norcocaine nitroxide [14,15]. Norcocaine nitroxide increases lipid peroxidation, causing mitochondrial swelling, eventually leading to cell death [15]. Cocaine also depletes glutathione, an important antioxidant involved in the hepatic metabolism of many drugs and protector against oxidative stress [8]. 

N-acetylcysteine, a drug used for acetaminophen overdose, has been shown to protect against cocaine-induced hepatic damage in mice-model studies [8]. By replenishing glutathione, NAC protects against the creation of free radicals and hepatotoxic compounds [1,8]. N-acetylcysteine also causes vasodilation by increasing nitric oxide. This drug-mediated vasodilation can help offset the effects of cocaine-induced abdominal shock by increasing hepatosplanchnic blood flow [11]. Because of these dual mechanisms, NAC is used as a mucolytic and adjuvant therapy in the treatment of chronic obstructive pulmonary disease, influenza, idiopathic pulmonary fibrosis, and contrast-induced nephropathy [6]. We hypothesize that NAC helped reverse our patient’s cocaine-induced liver failure by increasing hepatic perfusion and replenishing the protective antioxidant glutathione. This would account for the rapid change in the clinical picture from day one of administration of NAC, when the patient was intubated and required vasopressors, to day two of NAC, when the patient was extubated, and vasopressors were discontinued because he was hemodynamically stable. The only other medications the patient was administered during this time were lactulose and rifaximin, which do not systemically increase perfusion, but instead increase metabolism and excretion of ammonia.

We hypothesize that systemic vasoconstriction also accounts for the incidental finding of acute pancreatitis in our case. Acute pancreatitis is diagnosed when two of the following criteria are met: abdominal pain radiating to the back, elevated amylase and lipase two to three times the normal limit, and imaging that shows pancreatic inflammation [16]. While our patient had CT imaging that showed acute pancreatic inflammation, his serum lipase was within normal limits. As the patient was unable to fully participate in the exam due to his progressive hepatic encephalopathy, abdominal pain radiating to the back, and thus acute pancreatitis, could not be ruled out. 

Acute pancreatitis is most commonly due to alcohol use or gallstones obstructing the common bile duct [17]. Drug-induced acute pancreatitis is a rarer etiology and is a diagnosis of exclusion [17]. We were able to exclude alcohol use and gallstones in our patient through his negative blood alcohol levels and negative imaging studies. This suggested that our patient’s pancreatitis was a sequelae of cocaine-induced hypoperfusion.

Cocaine-induced acute pancreatitis is very rare. Cocaine is believed to cause acute pancreatitis by vasoconstriction and decreased mesenteric perfusion, which both lead to ischemia and inflammation of the pancreas [16]. This inflammation likely occurred secondarily in our patient due to the shared blood supply of the liver and the pancreas. The liver and the pancreas share the common hepatic artery before branching into their respective blood supplies [18]. Vasoconstriction of the common hepatic artery would reduce blood flow to the proper hepatic artery and the anterior and posterior superior pancreaticoduodenal arteries that supply the head of the pancreas, likely contributing to pancreatitis.

This systemic complication manifested in our patient as periumbilical ecchymoses, also known as Cullen’s sign [19]. It is a sign of retroperitoneal hemorrhage and is postulated to be caused by the lymphatic extravasation of inflammatory products and blood into the subcutaneous tissue surrounding the umbilicus [19]. While Cullen's sign is associated with acute pancreatitis, it is not specific to this disorder and may also indicate a ruptured ectopic pregnancy or rectus sheath hematoma [19]. In our patient, this finding was confirmed to be associated with acute pancreatitis through the patient’s CT results. 

Cocaine-induced acute liver failure and pancreatitis are not common in our patient population and add a new presentation to the differential diagnosis for patients presenting with signs of hepatic encephalopathy. According to the 2023 Alabama Drug Threat Assessment, cocaine is ranked fourth highest in the state for greatest drug threat after fentanyl and other opioids, methamphetamine, and heroin [20]. However, it is ranked sixth behind cannabis and prescription opioids in terms of patients seeking treatment for drug use disorder [20]. Even though cocaine use may not seem as urgent a health concern as some other drug misuse in Alabama, it is widely available and is at an increased risk of being mixed with other drugs or impure compounds prior to being sold to users [20]. During the month our patient presented to the emergency room, our inpatient service treated two other patients with life-threatening cocaine-induced illnesses. While they were not of the same etiology, they occurred in the same region and likely originated from the same inventory. We hypothesize that cocaine impurities could account for these atypical presentations of cocaine toxicity.

## Conclusions

After a review of the literature, we hypothesize that cocaine may cause acute liver failure through two mechanisms of injury: hypoperfusion-induced ischemia and increased oxidative stress due to the depletion of glutathione, both of which may be addressed through the utilization of N-acetylcysteine. Currently, available medical literature reports conflicting results on whether N-acetylcysteine is beneficial in non-acetaminophen-induced acute liver failure. However, in our case, the administration of N-acetylcysteine improved the patient’s liver function tests and coagulation studies, eliminating the need for a liver transplant. More studies should be conducted in the future on the potential benefit of this medication when used in cocaine-induced acute liver failure.
